# Identification of a Novel *bla*_NDM_ Variant, *bla*_NDM-33,_ in an Escherichia coli Isolate from Hospital Wastewater in China

**DOI:** 10.1128/mSphere.00776-21

**Published:** 2021-10-13

**Authors:** Tao Wang, Yuan Zhou, Chunhong Zou, Zhichen Zhu, Jie Zhu, Jingnan Lv, Xiaofang Xie, Liang Chen, Siqiang Niu, Hong Du

**Affiliations:** a Department of Clinical Laboratory, The Second Affiliated Hospital of Soochow University, Suzhou, Jiangsu, China; b Department of Ophthalmology, The Second Affiliated Hospital of Soochow University, Suzhou, Jiangsu, China; c Department of Laboratory Medicine, The First Affiliated Hospital of Chongqing Medical University, Chongqing, China; d Hackensack Meridian Health Center for Discovery and Innovation, Nutley, New Jersey, USA; e Department of Medical Sciences, Hackensack Meridian School of Medicine, Nutley, New Jersey, USA; Antimicrobial Development Specialists, LLC

**Keywords:** CRE, *E. coli*, *bla*_NDM-33_, hospital sewage

## Abstract

Since the discovery of NDM-1 and the worldwide reporting of different variants have raised alarms concerning global health, the problem of carbapenem-resistant *Enterobacterales* (CRE) has become increasingly serious. Therefore, research on the hydrolytic activity and molecular structure of NDM variants is beneficial to the development of antibacterial drugs. NDM has been evolving into variants that possess different hydrolysis activities toward β-lactam antibiotics. Here, we characterized a novel *bla*_NDM_ variant, named *bla*_NDM-33_, identified from a multidrug-resistant Escherichia coli strain from hospital sewage. NDM-33 differed from NDM-5 with a single-amino-acid substitution (A72T). *bla*_NDM-5_ was located in the Tn*125*-related *bla*_NDM-33_ region from an IncX3-type plasmid, pHD6415-NDM, that can be transferred horizontally. The genetic construct of *bla*_NDM-33_ showed higher MICs of carbapenems than a *bla*_NDM-5_ construct. Enzyme kinetics showed that NDM-33 had higher enzymatic activity for meropenem and cefazolin than NDM-5. The emergence of this novel NDM variant could pose a threat to public health because of its transferability and enhanced carbapenem activity.

**IMPORTANCE** Our study described a novel NDM-33 variant from an E. coli strain isolated from hospital sewage, where it was associated with human disease and antibiotic exposure. Importantly, hospital sewage was increasingly considered to be related to CRE hosts. Pathogens were transmitted from reservoirs through direct and indirect contact, ingestion, and inhalation of contaminated water or aerosols. In addition, under the selective pressure of antibiotics, NDM variants will become the main strain in the hospital water system and evolve into high virulence and high resistance. The monitoring of NDM mutants is of great significance for preventing and controlling the evolution of superbugs.

## OBSERVATION

The prevalence of carbapenem-resistant *Enterobacterales* (CRE) has increased since the early 2000s, representing a tremendous public health threat (1). NDM-1 was first detected in a Klebsiella pneumoniae isolate from a Swedish patient with urinary tract infection who traveled to New Delhi in 2008 (2). Since then, 32 NDM variants have been described or the sequences have been deposited in the GenBank database (https://www.ncbi.nlm.nih.gov/). Among them, the NDM-5 variant was first found in a multidrug-resistant Escherichia coli ST648 isolate recovered from the perineum and throat of a patient in the United Kingdom, and it showed enhanced hydrolytic activity compared with NDM-1 (3). In this study, we described a novel *bla*_NDM-33_ variant, identified from a multidrug-resistant E. coli strain, HD6415, from a hospital sewage sample.

The E. coli strain HD6415 was isolated from the sewage of the second affiliated hospital of Soochow University (Suzhou, China) in July 2019. We used LB plates containing 0.5 mg/liter meropenem for primary screening of sewage separated from each department in the hospital to study the distribution of CRE and further follow-up research. Bacterial antimicrobial susceptibility testing was performed using the broth microdilution method, and the results were interpreted according to the 2020 Clinical and Laboratory Standards Institute (CLSI) guidelines (4). The EUCAST (http://www.eucast.org/) breakpoints were used for colistin and tigecycline. The testing was performed in triplicates in two different days, and Escherichia coli ATCC 25922 was used as the quality control (QC) strain.

The strain HD6415 was resistant to most of the tested antimicrobial agents, including carbapenems (ertapenem, imipenem, and meropenem), ciprofloxacin, ceftazidime, and ceftazidime-avibactam. MICs of meropenem, ertapenem, and imipenem for HD6415 were 512, 256, and 512 mg/liter, respectively (Table 1). HD6415 was only susceptible to kanamycin (MIC = 8 mg/liter), tigecycline (MIC = 1 mg/liter), colistin (MIC = 2 mg/liter), and aztreonam (MIC = 4 mg/liter).

**TABLE 1:** Antimicrobial drug susceptibility profile

Drug	MIC (mg/liter) for:				
	HD6415 (parental strain)	EC600/pHD6415-NDM (transconjugant)	DH5α/pET28a-*bla*_NDM-5_ (*bla*_NDM-5_ construct)	DH5α/pET28a-*bla*_NDM-33_ (*bla*_NDM-33_ construct)	DH5α/pET28a(empty vector)
Meropenem	512	64	32	64	0.125
Imipenem	512	256	256	512	0.125
Ertapenem	256	64	32	64	0.25
Ceftazidime	512	256	512	512	0.5
Aztreonam	4	0.125	0.125	0.125	0.125
Ceftazidime/Avibactam	512/4	256/4	256/4	64/4	0.25/4
Ciprofloxacin	32	0.125	0.125	0.125	0.125
Kanamycin	8	2	32	32	32
Tigecycline	1	0.5	0.5	0.5	0.5
Colistin	2	2	2	2	1

The presence of *bla*_NDM_ was initially determined by PCR (using primers ATGGAATTGCCCAATATTATGC [F] and TCAGCGCAGCTTGTCGG [R]) (5). Next-generation sequencing of the HD6415 genome was conducted using a paired-end library with an average insert size of 350 bp on a NovaSeq 6000 platform (Illumina, CA, USA). The raw reads were assembled *de novo* using SPAdes v3.11 (http://cab.spbu.ru/software/spades/). Further plasmid assembly was obtained by mapping contigs on reference sequences, checking overlapping paired ends, and confirming the assembly by the PCR-based gap closure method. The acquired antimicrobial resistance genes were identified using ResFinder 4.0 (6). *In silico* multilocus sequence typing (MLST; https://cge.cbs.dtu.dk/services/MLST/) showed HD6415 belonged to ST650. HD6415 contains genes encoding resistance to aminoglycosides [*aac*(3)*-IIa*, *aadA1*, and *aadA5*], β-lactams (*bla*_OXA-1_, *bla*_NDM-33_, and *bla*_TEM-1_), phenicols (*floR* and *catA1*), tetracycline [*tet*(A) and *tet*(B)], fluoroquinolone (*qepA2* and *qnrS2*), and macrolide, lincosamide, and streptogramin B (MLS) [*mdf*(A)]. The mutations of *gyrA* (D87N; S83L) and *parC* (S80I) lead to the isolate being resistant to ciprofloxacin.

The *bla*_NDM-33_ variant showed a single-nucleotide difference (G214A) compared with *bla*_NDM-5_, resulting in an amino acid substitution at codon 72 (A72T). The A72T amino acid substitution in NDM-33 was in the first active-site ring between the β2 and β3 chains, which shifted away from the Zn center to accommodate substrates with different molecular structures (7). Previous studies showed that base substitutions in this region did not affect the overall folding of the protein, but different thermal stabilities have been observed in NDM variants. NDM variants with double amino acid substitutions, e.g., NDM-8 (D130G, M154L), NDM-5 (V88L, M154L), and NDM-7 (D130N, M154L), appeared to be more stable to thermal denaturation than the singly substituted NDM-6 (A233V), NDM-3 (D95N), and NDM-4 (M154L) (8).

**FIG 1:**
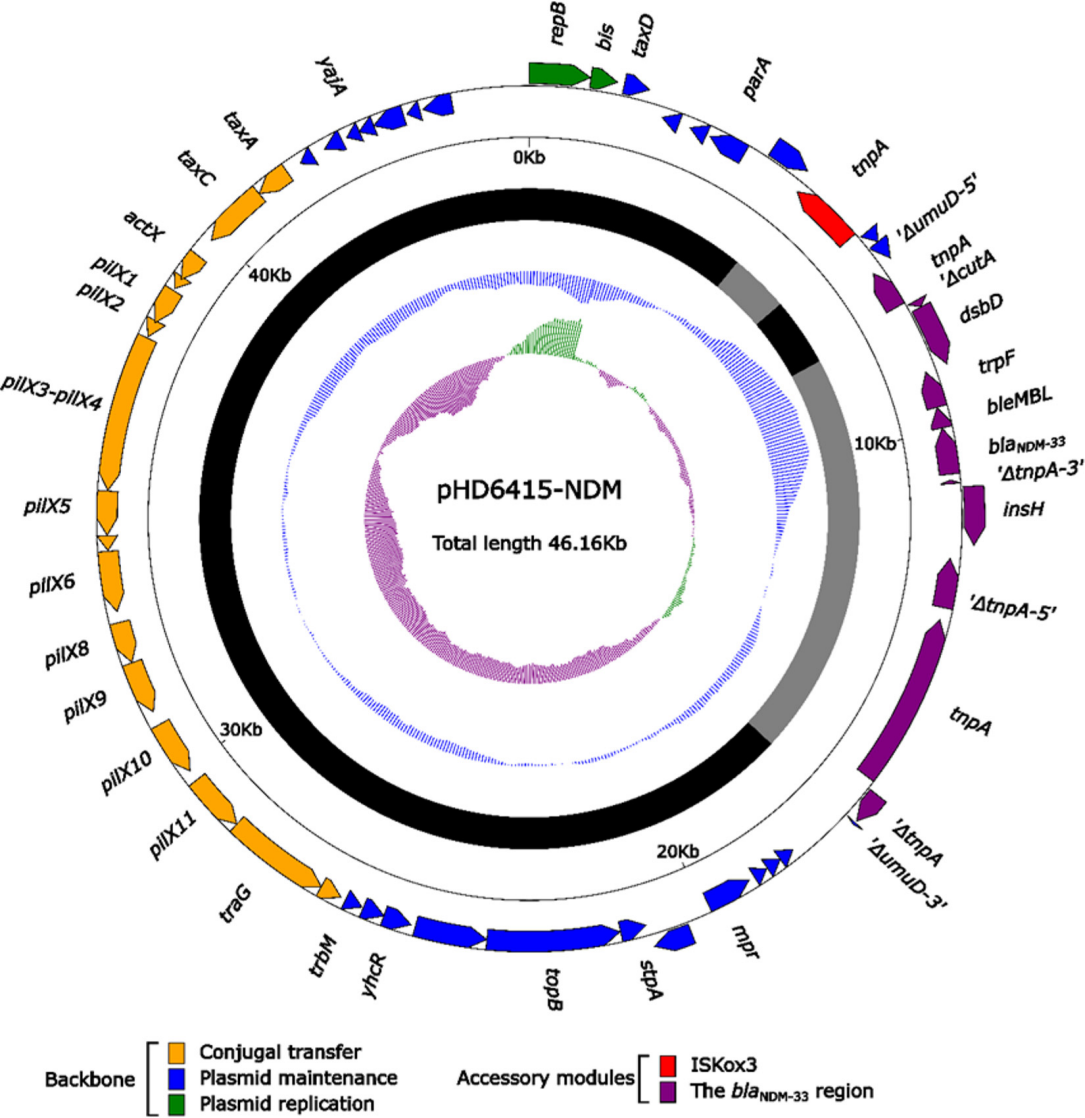
Schematic diagram of the plasmid pHD6415-NDM. Genes of different functions are denoted by arrows and presented in various colors. The circles show (from outside to inside) predicted coding sequences, scale in 10 kb, backbone (black), and accessory module (gray) regions, GC content, and GC skew [(G–C)/(G+C)].

**FIG 2:**
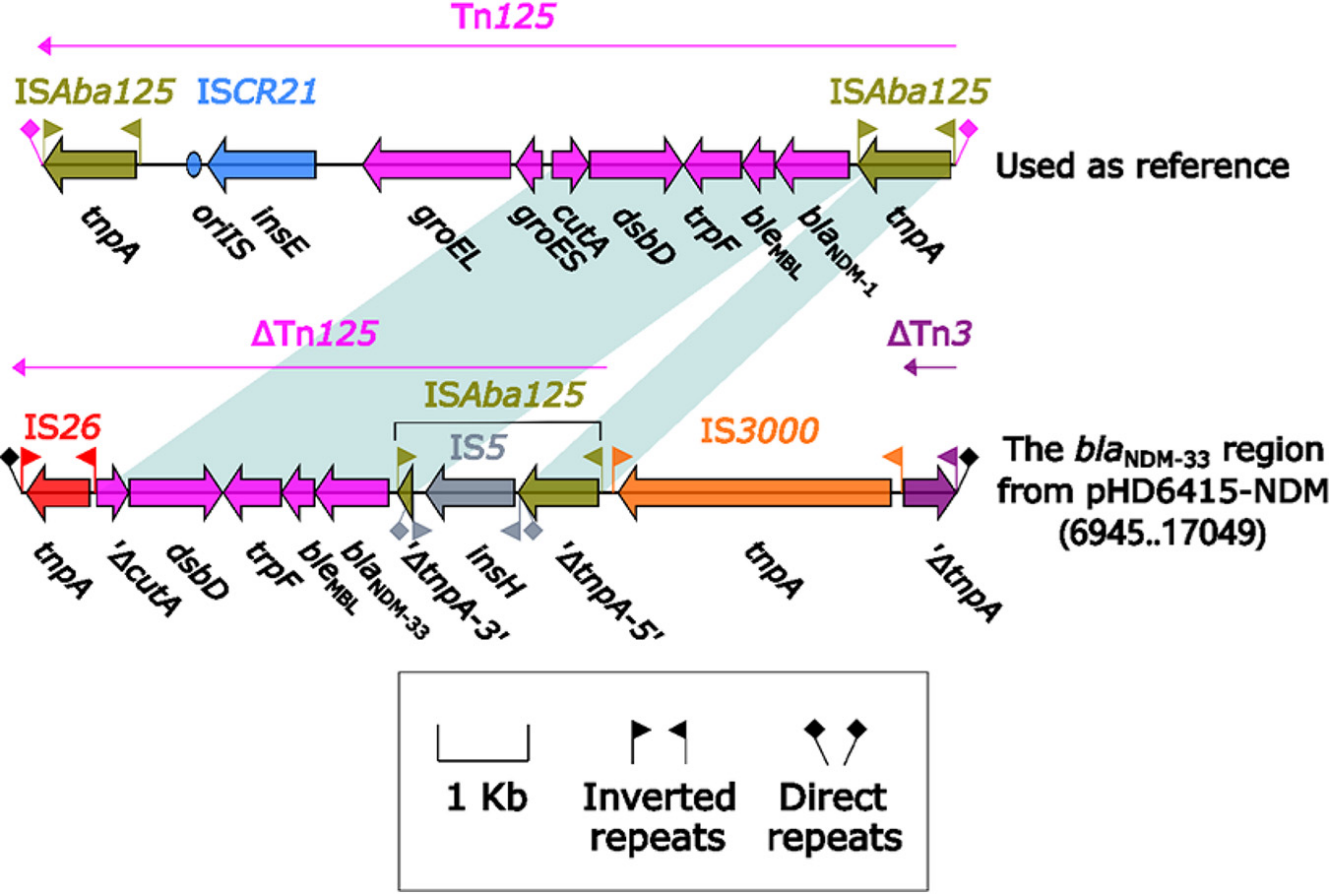
Linear comparison of the *bla*_NDM-33_ region and related Tn*125*. Genes are denoted by arrows. Genes, mobile elements, and other features are colored based on their functional classification. Shading denotes regions of homology (nucleotide identity, ≥95%). Numbers in parentheses indicate nucleotide positions within the plasmid pHD6415-NDM. The accession number of Tn*125* used as the reference is JN872328 (14).

Currently, *bla*_NDM_ has been rapidly spreading in more than 40 countries worldwide through plasmids in different replicon types, including IncX3, IncF, IncN, IncA/C, IncR, and IncT (9, 10). Among them, IncX3 plasmid was a major vehicle in mediating the dissemination of *bla*_NDM_ in China (11). The genome sequence analysis showed *bla*_NDM-33_ was also carried by a 46.16-kb IncX3-type plasmid (pHD6415-NDM) (Fig. 1). *bla*_NDM-33_ was the only antimicrobial resistance gene presented on pHD6415-NDM, which was almost identical (100% query coverage and >99.9% nucleotide identities) to numerous IncX3-type plasmids carrying *bla*_NDM_, such as pNDM5 (carrying *bla*_NDM-5_; GenBank accession number KU761328), pJN05NDM7 (carrying *bla*_NDM-7_; GenBank accession number MH523639), pNDM-20 (carrying *bla*_NDM-20_; GenBank accession number MF458176), and pNDM21_020023 (carrying *bla*_NDM-21_; GenBank accession number CP025948). Similar to the other *bla*_NDM_-harboring IncX3 plasmids, *bla*_NDM-33_ was located in a Tn*125*-related element (Fig. 2), which was inserted within the backbone gene *umuD* of pHD6415-NDM and bracketed by the 3-bp direct repeats (DRs; target site duplication signals for transposition). This region was composed of multiple mobile genetic elements, including IS*26*, ΔTn*125*, IS*3000*, and ΔTn*3*.

To investigate the transferability of pHD6415-NDM, a conjugation experiment was carried out using the rifampin-resistant E. coli strain EC600 as the recipient. The transconjugant was selected on an LB agar plate containing 4 mg/liter meropenem and 200 mg/liter rifampin, and then the presence of *bla*_NDM-33_ in the transconjugant was confirmed by PCR and Sanger sequencing. Results showed *bla*_NDM-33_ can be successfully transferred via conjugation, with an efficiency of ∼1.2 × 10^−2^ (transconjugant/recipient). Susceptibility testing showed that the transconjugant had meropenem, imipenem, and ertapenem MICs of 64, 256, and 64 mg/liter, respectively, in consistent with the carbapenem resistance phenotype observed in the parental strains (Table 1).

To further evaluate whether the A72T substitution in NDM-33 confers different levels of resistance to β-lactam antibiotics, we cloned the full-length *bla*_NDM-33_ and *bla*_NDM-5_ along with their identical natural promoters into the pET28a vector, followed by transformation into E. coli DH5α cells. Meanwhile, the pET28a plasmid was transformed as a control (12). Susceptibility testing results showed that the carbapenem (ertapenem, imipenem, and meropenem) MICs in the *bla*_NDM-33_ construct (DH5α/pET28a-*bla*_NDM-33_) were 2-fold higher than those in the *bla*_NDM-5_ construct (DH5α/pET28a-*bla*_NDM-5_) (Table 1).

Steady-state enzyme kinetic parameters were performed for NDM-5 and NDM-33 as described previously (13). Briefly, the *bla*_NDM-5_ and *bla*_NDM-33_ gene sequences without the peptide signal region were amplified using primers EcoRI-NDM-F (5′-CCGGAATTCATGGAATTGCCCAATAT-3′) and HindIII-NDM-R (5′-CCCAAGCTTTCAGCGCAGCTTGTCGGCC-3′), followed by cloning into the pET28a plasmid and transforming into E. coli BL21. The NDM-5 and NDM-33 enzymes were purified and suspended in the HEPES buffer (pH 7.5), containing 250 mM NaCl, 100 μM ZnCl_2_, 20 μg/ml bovine serum albumin, at 25°C. The real-time absorbances of meropenem (298 nm), imipenem (297 nm), ceftazidime (257 nm), aztreonam (318 nm), cefotaxime (264 nm), cefepime (254 nm), piperacillin (232 nm), cefazolin (270 nm), ceftriaxone (240 nm), and ampicillin (235 nm) were determined under initial-rate conditions with a Shimadzu UV2550 spectrophotometer (Kyoto, Japan). The results showed that NDM-33 could hydrolyze all tested β-lactams except aztreonam (Table 2). The *k*_cat_/*K*_m_ ratios for meropenem and cefazolin of NDM-33 were higher than those of NDM-5, but ceftazidime and cefepime *k*_cat_/*K*_m_ ratios were lower than those of NDM-5. These results suggested that NDM-33 had higher enzymatic activity against meropenem and cefazolin and lower activity against ceftazidime and cefepime relative to NDM-5.

**TABLE 2:** Steady-state enzyme kinetics of NDM-5 and NDM-33

Drug	NDM-5			NDM-33		
	*K*_m_ (μM)	*k*_cat_ (S^−1^)	*k*_cat_/*K*_m_ (μM^−1^·S^−1^)	*K*_m_ (μM)	*k*_cat_ (S^−1^)	*k*_cat_/*K*_m_ (μM^−1^·S^−1^)
Ampicillin	98	337.07	3.42	82	47.85	0.58
Imipenem	73	278.50	3.81	53	177.23	3.32
Meropenem	68	133.13	1.96	18	58.81	3.24
Cefazolin	163	84.97	0.52	64	266.18	4.14
Cefotaxime	38	146.68	3.81	32	64.80	2.05
Ceftazidime	30	107.71	3.58	68	77.85	1.15
Cefepime	34	77.34	2.29	103	54.48	0.53
Aztreonam	NH^*a*^	NH	NH	NH	NH	NH

a NH, not detectable due to a low initial rate of hydrolysis.

Taken together, our study described a novel NDM-33 variant from an E. coli strain isolated from hospital sewage. Hospital wastewater serves as an important reservoir for the emergence and transmission of antimicrobial resistance genes and variants, although it represents an environmental sample in which a high density of antibiotic residues and antibiotic-resistant bacteria are present. To some extent, the phenotypic differences between NDM-5 and NDM-33 are not remarkable. However, the continuous evolution of NDM enzymes could foster the emergence of novel variants that possess different hydrolysis activities toward β-lactam antibiotics. Our study enriched our understanding of enzymatic function and evidenced the ongoing evolution of NDM enzymes. A close surveillance of NDM-producing bacteria, both environmentally and clinically, should be enacted to monitor and control the spread of NDM variants.

### Data availability.

The draft whole-genome sequence of strain HD6415 was submitted to GenBank under accession number JAGTHW000000000, and the complete sequence of plasmid pHD6415-NDM was submitted to GenBank under accession number MZ004933.
